# Burden of Depressive disorders in Balkan countries

**DOI:** 10.1093/eurpub/ckac130.189

**Published:** 2022-10-25

**Authors:** J Todorovic, M Santric-Milicevic, A Stevanovic, Z Terzic-Supic, Z Stamenkovic, T Albreht, S Stoisavljevic, N Terzic, L Scepanovic

**Affiliations:** 1 Institute of Social Medicine, Medical Faculty, University of Belgrade, Belgrade, Serbia; 2 Institute of Public Health of Slovenia, Ljubljana, Slovenia; 3 Institute of Public Health of Republika Srpska, Banja Luka, Bosnia and Herzegovina; 4 Faculty of Medicine, Banja Luka, Bosnia and Herzegovina; 5 Institute of Public Health of Montenegro, Podgorica, Montenegro

## Abstract

**Background:**

Depressive disorders are one of the leading causes of disability and are significant contributor to total Global Burden of Disease (GBD). Globally, according to the 2019 GBD study, the 1.85% of all Disability adjusted life years (DALYs) was associated with depression. The aim of this study was to describe the age-standardized DALYs in ten Balkan countries and the changes observed between 1990 and 2019.

**Methods:**

The study included the data on age-standardized DALY rate per 100,000 for depressive disorders in the period between 1990 and 2019 for ten Balkan countries (Albania, Bosnia and Herzegovina, Bulgaria, Croatia, Greece, Montenegro, North Macedonia, Romania, Serbia and Slovenia) from the Institute of Health Metrics and Evaluation. We acknowledge the support from the COST Action 18218 - European Burden of Disease Network.

**Results:**

The highest age-standardized DALY rate/ 100,000 throughout the observed period was in Greece in the 1990- 914.48 (95% CI: 620.19-1287.16) and 927.75 (95% CI: 626.5-1295.7) in 2019, while the lowest age-standardized DALY rate per 100,000 was in Albania in the entire observed period (1990- 369.032, 95% CI: 253.52-518.04 and 2019- 386.42, 95% CI: 266.46- 536.31). Greece and Albania are only two countries in the Balkans in which the age-standardized DALY rate per 100,000 increased in the period between 1990 and 2019, in all other countries there was a slight decrease in age-standardized DALY rates in the period between 1990 and 2019.

**Conclusions:**

Age-standardized DALY rates per 100,000 decreased in eight out of ten countries in the Balkan region. However, age-standardized DALY rate per 100,000 increased in the country with the highest burden of depressive disorders in the region- Greece. In the period between 1990 and 2019 the lowest burden of depressive disorders measured in age-standardized DALY rate /100,000 was in Albania, but Albania also recorded the increase in burden.

**Key messages:**

- Burden of depressive disorders in Balkan countries is significant.
- Key public health stakeholders in Greece should address the increasing trend of DALY and its association with economic crisis.

